# Effects of chocolate milk consumption on markers of muscle recovery following soccer training: a randomized cross-over study

**DOI:** 10.1186/1550-2783-7-19

**Published:** 2010-05-18

**Authors:** Stephanie F Gilson, Michael J Saunders, Charles W Moran, Rebecca W Moore, Christopher J Womack, M Kent Todd

**Affiliations:** 1 Department of Kinesiology, MSC 2302, James Madison University, Harrisonburg, VA 22807, USA; 2 Department of Kinesiology, Michigan State University, 134 Intramural Sports Circle, East Lansing MI 48824-1034, USA

## Abstract

**Background:**

The efficacy of chocolate milk (CM) as a recovery beverage following a period of increased training duration (ITD) was studied in intercollegiate soccer players.

**Methods:**

13 subjects completed one week of normal 'baseline' training followed by four days of ITD. After each day of ITD, subjects received either a high-carbohydrate (504 kcal; CHO: 122 g; 2 g Fat) or isocaloric CM (504 kcal; 84 g CHO; 28 g Pro; 7 g Fat) recovery beverage. Serum creatine kinase (CK), myoglobin (Mb), muscle soreness, fatigue ratings and isometric quadriceps force (MVC) were obtained prior to ITD, and following 2- and 4-days of ITD. Performance tests (T-drill, vertical jump) were performed within training sessions. Treatments were administered in a randomly counterbalanced protocol, and subjects repeated the procedures with the alternate beverage following a two-week washout period.

**Results:**

Mean daily training time and HR increased (p < 0.05) between baseline training and ITD, with no differences between treatments. No treatment*time effects were observed for Mb, muscle soreness, fatigue ratings and MVC. However, serum CK was significantly lower (p < 0.05) following four days of ITD with CM (316.9 ± 188.3 U·L^-1^) compared to CHO (431.6 ± 310.8 U·L^-1^). No treatment differences were observed for the performance tests.

**Conclusions:**

Post-exercise CM provided similar muscle recovery responses to an isocaloric CHO beverage during four-days of ITD. Future studies should investigate if the attenuated CK levels observed with CM have functional significance during more demanding periods of training.

## Background

The timing and composition of nutrient intake can significantly influence recovery from heavy exercise (i.e. [1-10]). Increased carbohydrate intake immediately following exercise results in faster rates of muscle glycogen replenishment [1,2] and can attenuate symptoms of overreaching during periods of intensified endurance training, such as negative mood states, increased perceived exertion, and impaired performance [3]. The addition of protein to post-exercise carbohydrate feedings can also influence recovery from heavy exercise. Carbohydrate and protein (CHO+Pro) supplementation has been shown to attenuate markers of sarcolemmal disruption, such as creatine kinase (CK) and myoglobin [4-10], reduce muscle soreness [6,7,11] and improve subsequent muscle function [5,10] compared to carbohydrate-only beverages, though not all studies have reported these effects [11-13]. In addition, CHO+Pro ingestion during recovery from heavy exercise has been shown to improve performance in subsequent whole-body exercise in some [9,14-18], but not all studies [6-8,11,19-21].

Chocolate milk (CM) has been investigated recently as a potential recovery beverage, as it contains carbohydrate and protein in similar amounts to CHO+Pro beverages associated with improved post-exercise recovery. In addition, CM has been noted for its' good taste, wide availability, low cost and convenience, which could make it a popular alternative to commercial sports beverages. Two studies reported that CM consumption following a heavy endurance exercise session was associated with equal [22] or superior [23] performance during subsequent exercise compared to carbohydrate alone. Similarly, Cockburn et al. [5] reported that compared to carbohydrate beverages, CM ingestion during recovery from heavy eccentric exercise improved peak torque and total work during subsequent exercise. However, the carbohydrate beverages utilized in each of these studies contained fewer calories than CM, so it is possible that the purported benefits may have been related to caloric differences between treatments.

At least two studies have examined CHO+Pro ingestion in free-living endurance athletes. Luden et al. [6] reported that CHO+Pro attenuated plasma CK and muscle soreness compared to CHO in collegiate distance runners during six days of training. Similarly, Cade et al. [24] reported improvements in plasma CK and lactate dehydrogenase with CHO+Pro supplementation during intensive training in collegiate swimmers. However, we are aware of no studies comparing CHO and CHO+Pro treatments on recovery in team-sport athletes such as soccer players. Soccer is an alternating-intensity endurance sport which has been shown to significantly reduce muscle glycogen stores [25,26]. In addition, plyometric exercises such as those utilized in soccer training have been associated with increased muscle soreness, elevated blood CK levels and impaired performance in subsequent exercise [27]. Thus, the utilization of post-exercise nutrition interventions that influence these variables could potentially affect recovery in soccer players. The purpose of this study was to compare the effects of CM to an isocaloric carbohydrate beverage on markers of recovery following a period of increased training duration in competitive soccer players.

## Methods

### Participants

Twenty-two NCAA Division I male soccer players volunteered for the study following a complete explanation of procedures. Five subjects failed to complete all testing, or were unable to complete consistent training programs due to musculoskeletal injuries unrelated to the study. Four subjects were excluded from final statistical analyses due to large variations in dependent measurements between baseline periods (described below) resulting in 13 subjects included in data analyses. Prior to the study, all potential subjects signed an informed consent form and completed a Pre-participation Screening Questionnaire [28]. Individuals with preexisting injury, those taking medications to relieve soreness, or with milk allergies were excluded from study participation. All procedures were approved by the JMU Institutional Review Board prior to initiation of the study.

A minimum of 12 participants were recruited for the present study, in order to detect potential between-treatment differences of 1.2-1.6 SD units with a β > 0.80. This sample size was estimated using calculations from Lipsey [29], and utilized effect-sizes reported in previous studies comparing the effects of CHO+Pro and CHO beverages on the dependent measures utilized in this study (i.e. [7,9,10]). For example, using mean values reported by Valentine et al. [10], CHO+Pro ingestion produced an effect on post-exercise plasma CK values of approximately 1.6 SD units, assuming a correlation of 0.80 between repeated measurements [29].

### Training Protocols

All testing was conducted during the athletes' off-season training period. On two occasions, subjects performed one week of normal 'baseline' training, followed immediately by four days of increased training duration (ITD). Baseline training levels represented typical training types/amounts conducted by the team during off-season training. The ITD period was intended to increase total training duration by >25% during four consecutive days of training. The number of days of ITD (and daily training times) were selected to produce a practically-relevant increase in training demands, without violating NCAA regulations limiting Division I athletes outside of the playing season to a maximum of 8 hr of athletically-related activities per week (NCAA Playing and Practice Limitations, Bylaw 17.1.5.2).

Daily training sessions (Mon-Fri) consisted of alternating days of a) soccer-specific training drills and aerobic development activities, and b) strength and sprint training (Table 1). On Mon/Wed/Fri, the prescribed training sessions consisted of a) warm-up (~10 min), b) agility drills (~10 min), c) main training session, and d) cool down (~10 min). The length of the main training segment on these days varied from 60-90 min (depending on whether it occurred during baseline or ITD), and included soccer-specific training drills and game-play, with a heavy aerobic conditioning component. On Tu/Th the prescribed training consisted of a) warm-up (~10 min), b) main training session, and c) cool down (~10 min). The main training session on these days included sprint/plyometric training drills (such as 'ladder footwork', standardized agility runs and coordination drills), followed by resistance training exercises. The length of the main training segment varied from 55-70 min on these days (baseline or ITD). Sprint/plyometric exercises and resistance training comprised an equal portion of the main training session on these days. No organized training sessions were conducted for two days prior to the ITD periods (Sat/Sun). Athletes were permitted to exercise on their own, but were instructed to limit exercise to a maximum of 30-45 minutes of low-intensity aerobic exercise (jogging).

**Table 1:** Prescribed Training Regimen

		Length of Session	
Day of Training	Training Type	Week 1: Baseline	Week 2: ITD
Mon	Soccer-specific training drills and aerobic development	90 min	120 min
Tues	Strength and sprint training	75 min	90 min
Wed	Soccer-specific training drills and aerobic development	90 min	120 min
Thurs	Strength and sprint training	75 min	90 min
Fri	Soccer-specific training drills and aerobic development	90 min	N/A
Sat	Low-intensity aerobic recovery	30-45 min (unsupervised)	N/A
Sun	Low-intensity aerobic recovery	30-45 min (unsupervised)	N/A

Specific training exercises were prescribed to be virtually identical between the two treatment periods. Training variables were recorded throughout the exercise sessions to quantify exercise intensity, and to ensure consistency between training periods. Heart rate was obtained during all training sessions (but not recorded during resistance training exercises) using a Polar heart-rate monitor (Brooklyn, NY). Average heart rate values for each training session were recorded. Ratings of perceived exertion (RPE) were obtained using the Borg RPE 6-20 scale immediately after each training session. Total exercise time was also recorded for each training session.

Participants completed all procedures on two occasions, with a two-week period of recovery and resumed training between the two study periods. A randomly counterbalanced design was utilized so that any changes in dependent measurements over time would be randomly distributed within each treatment period. Each training session was conducted by the teams' coaches, under the supervision of the investigators.

### Physiological Measurements

The following measurements were obtained on Monday (Pre ITD), Wednesday (Post2), and Friday (Post4) of each ITD period. On these dates, subjects reported to the laboratory prior to the daily practice session, approximately 18-22 hours following the previous day's training session. The specific measurement time varied between subjects to accommodate individual schedules, but was scheduled at a consistent time over the course of the study for each subject. Measurements are listed below in the order in which they were obtained during testing sessions.

Muscle Soreness Ratings: Soreness ratings were obtained using a 100 mm visual analog scale, with 0 indicating no muscle soreness and 100 indicating impaired movement due to muscle soreness, as described previously [30]. Subjects were asked to describe their overall level of muscle soreness in the legs while performing normal daily activities such as walking up or down stairs.

Mental and Physical Fatigue Ratings: These ratings were obtained using Part II of the *Mental and Physical State and Trait Energy and Fatigue Scales *(MPSTEFS; P.J. O'Connor, personal communication). Separate ratings were obtained for Physical Energy, Physical Fatigue, Mental Energy and Mental Fatigue, on the basis of " how do you feel right now" instructions, as described by Kline et al. [31]. Each rating represented the combined scores from three visual analog scales of 0-100 mm (i.e. the total score for Physical Energy represented the combined scores of three scales which rated the participant's relative degree of "energy", "vigor" and "pep"). Thus, each of the four energy/fatigue ratings had potential scores varying from 0-300 mm. Higher scores on this scale represented higher degrees of the variable (i.e. a higher "Mental Fatigue" score represented a higher degree of mental fatigue).

Serum Creatine Kinase (CK): Blood was obtained from an antecubital vein following completion of the muscle soreness and MPSTEFS questionnaires. Whole blood was spun in a centrifuge at 7000 rpm to obtain serum, which was stored at -80°C, brought to room temperature (22°C) prior to analysis, and mixed through gentle inversion. Serum CK was analyzed using a Johnson and Johnson Vitro DT 6011 analyzer, according to the manufacture's instructions. All samples were run in duplicate, and mean values were recorded.

Serum Myoglobin (Mb): Serum Mb levels were assessed using commercially available ELISA kits (BioCheck, Inc.) according to the manufacturer's instructions. A standard curve was prepared using reference standards ranging from 0 to 1,000 ng/mL Mb. Absorbance of the 96-well assay plate was read at a wavelength of 450 nm using a microplate spectrophotometer. All samples were run in duplicate on the same assay plate and mean values recorded.

Maximal Voluntary Contraction (MVC): Voluntary isometric peak force of the right quadriceps was assessed using a custom-built muscle function device. All subjects performed the test in an upright seated position with the right leg positioned at approximately 70° of knee flexion. Subjects provided a maximal 3 s leg extension against a stationary bar positioned at a standardized position on the shin. The right leg was used for all subjects (as opposed to dominant leg) to insure identical positioning of the shin against the stationary bar. Force measurements were obtained from a force transducer throughout each contraction, and peak force was obtained from each trial using custom designed software. Subjects performed three maximum voluntary contractions, with 1 min rest between trials. Peak force was recorded as the highest value from the three trials. Using the same testing protocols, we have previously observed a coefficient of variation (CV) of 6.9% between repeated trials performed under similar exercise conditions (i.e. male athletes tested prior to exercise with repeated trials separated by ~1 week).

### Performance Measurements

The following soccer-specific tests of performance were conducted on the dates indicated during the ITD periods.

Modified pro-agility test (T-drill): The test consisted of four directional changes (2 of 90 degrees, 2 of 180 degrees) in a 40 meter sprint test [32]. The test was completed on a grass field on Tuesday of the ITD periods, immediately prior to the start of strength training. Three testers recorded time to completion using manual digital stopwatches. Inter-tester variability was very low between these measurements (CV < 1%). The average of the three times was recorded for each trial. Each subject completed the test twice and the fastest trial time was recorded.

Vertical jump test: The test was performed on Friday of the ITD period. Subjects completed three vertical jumps, measured using a Vertec™ vertical jump assessment device with 0.5 inch increments. Countermovement jumps were performed for all trials, as described by Byrne and Eston [33]. Subjects were permitted to utilize their arms in the movement. The highest jump height of the three trials was recorded for each subject.

### Treatments and Dietary Controls

Immediately following each training session of the ITD period, subjects consumed one of two recovery treatment beverages described below. Specific treatments were assigned to the subjects using a randomly-counterbalanced design. Beverages were consumed within 5 minutes of completion of each exercise session.

Low-Fat Chocolate Milk Beverage (CM): Each serving consisted of 672 ml of CM, containing 84 g CHO, 28 g protein, 7 g fat, and approximately 504 total kcal (Table 2). Thus, each serving provided approximately 1.1 g CHO·kgBW^-1^, which approximates levels associated with optimal recovery of muscle glycogen [34,35].

**Table 2:** Comparison of Beverage Ingredients

Nutrient	CM	CHO
Volume (mL)	672	672
Energy (kcal)	504	504
Carbohydrate (g)	84	122
Protein (g)	28	0
Fat (g)	7	2
Sodium (mg)	511	277
Potassium (mg)	0	202
Vitamin C (mg)	7	302
Vitamin E (mg)	0	101
Calcium (mg)	852	101

Carbohydrate Beverage (CHO): Each serving provided 672 ml of an 18.6% carbohydrate beverage (~1.5 g CHO·kgBW^-1^), providing 122 g CHO, 0 g protein, 2 g fat, and approximately 504 total kcal (Table 2). Chocolate-flavored commercially-available carbohydrate gels (Clif Shots^®^) were mixed with water to provide similar taste and color to the CM beverage.

Subjects were assigned their beverage treatment order by a laboratory assistant who was not directly involved in the study, via a coin-flip. Once half of the participants had been assigned one of the beverages for their first treatment period (either CM or CHO), any remaining subjects were assigned the alternative beverage, to insure a counterbalanced allocation of treatments. Beverage preparation and labelling was conducted by an investigator who did not participate in the data collection process. Researchers were not aware which beverages the subjects were receiving until the study was completed. Similarly, the subjects were not informed of the composition of the beverages until cessation of the study. Anecdotal reports from subjects following the study suggest that subjects were aware of differences in taste between the beverages, but had no preconceived notions regarding differing ingredients or perceived efficacy. However, no systematic data was collected regarding subject perceptions of the beverages.

Subjects were instructed not to consume any other nutrients for 2-hours following each training session. Subjects were not required to adjust their regular diets (other than the post-exercise treatments they received), but were encouraged to replicate the same dietary habits during the two treatment periods. Dietary records were obtained for the four-day ITD period, and analyzed by FoodWise software (McGraw-Hill Science/Engineering/Math, 2005) for total caloric, protein, and fat intake during the periods of increased training volume.

### Statistical Analysis

Statistical testing was conducted using SPSS version 17.0 (Thomson Learning, Pacific Grove, CA), using an alpha level of p < 0.05 for all analyses. Training variables (average daily training time, heart rate and RPE) were analyzed using Repeated Measures Analysis of Variance (RM-ANOVA), with treatment (CM, CHO) and training period (baseline, ITD) as within-subject factors. Vertical jump performance and nutrient intake (carbohydrate, protein, fat) were compared between treatment periods using dependent t-tests. T-drill performance data was not normally distributed, and was therefore analyzed between treatments using a (non-parametric) Wilcoxon Signed Ranks test.

Most of the recovery variables (muscle soreness, MVC and all MPSTEFS ratings) were analyzed using RM-ANOVA, with treatment (CM, CHO) and time (PreITD, Post2, Post4) as within-subject factors. Post-hoc tests were conducted (where appropriate) to assess differences between individual time-points, with Bonferroni adjustments for multiple comparisons. Data for CK and Mb were not normally distributed, and thus were analyzed between treatments (at each time-point) using Wilcoxon Signed Ranks tests. Adjustments were made for multiple comparisons by dividing the alpha level by the number of comparisons for each variable.

Preliminary statistical analyses were performed on 17 subjects who completed all testing. However, some subjects exhibited large variances in baseline (PreITD) measurements between the two treatment periods, possibly due to activities outside of the study during the two unsupervised days prior to PreITD. This resulted in significant group differences in numerous PreITD measurements. In order to simplify interpretation of the hypothesis tests, absolute criteria were established to identify and remove individual subjects who exhibited large differences in PreITD values. These criteria were established using natural breaks in the score distributions. Four subjects exceeded the established criterion scores, and were thus eliminated from further statistical analyses. The exclusion criteria had the intended effect of eliminating all significant differences in PreITD values between treatments, making interpretation of the data simpler. However, it should be noted that exclusion of these subjects did not alter the outcomes of any hypothesis testing (i.e. any significant between-treatment outcomes reported in this manuscript were also significant with the entire group of 17 subjects).

## Results

### Participants

Statistical analyses were conducted on data from 13 collegiate NCAA Division I male soccer players. Average (± SEM) age, height and weight of the participants were 19.5 ± 0.3 y, 1.84 ± 0.02 m, and 79.4 ± 2.6 kg, respectively.

### Training Periods

Data obtained from the training sessions are provided in Table 3. Average daily training time and heart rate were significantly increased (p < 0.05) between the baseline and ITD periods. No differences in average training time, RPE or HR were observed between CHO and CM treatment periods. In addition, no significant differences (p > 0.05) in dietary intake (kcal, carbohydrate, protein, fat) were observed between training periods (data not shown, as only seven subjects provided complete records for both training periods).

**Table 3:** Daily Averages in Training Data

Baseline Training Period	CHO	CM
Time (min)	85.1 ± 1.4	85.5 ± 1.4
RPE (6-20)	13.7 ± 0.3	13.8 ± 0.2
HR (bt/min)	143 ± 3.4	141 ± 3.3

**Increased Training Duration**		

Time* (min)	95.5 ± 3.0	95.2 ± 1.4
RPE (6-20)	14.3 ± 0.4	13.8 ± 0.5
HR* (bt/min)	147 ± 3.0	143 ± 3.0

Data reported are Mean ± SEM, averaged for Monday through Thursday of each training week. * = Significantly greater than baseline (p < 0.05)

#### Recovery Variables & Performance Tests

**Figure 1:**
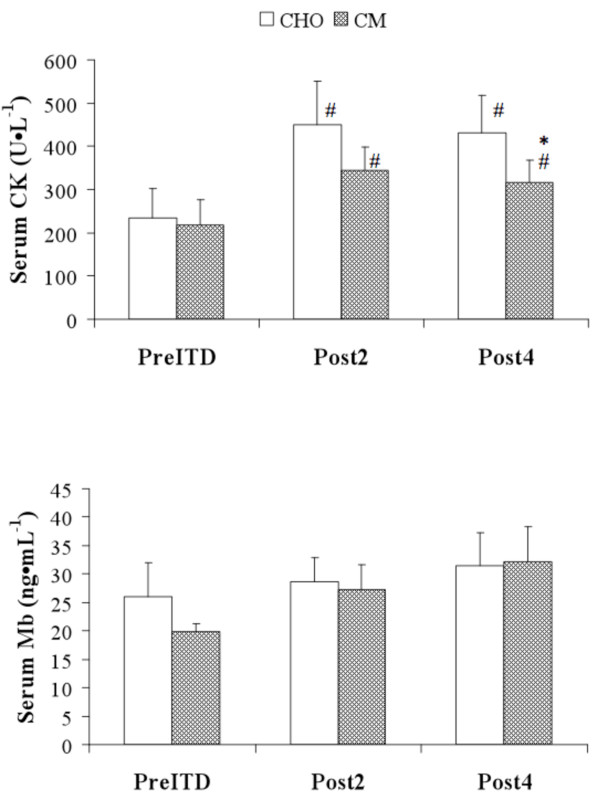
**Serum CK and Mb levels following Increased Training Duration**. Data reported are means/standard error. [* = significantly different (p < 0.05) than CHO; # = significantly different than PreITD].

**Figure 2:**
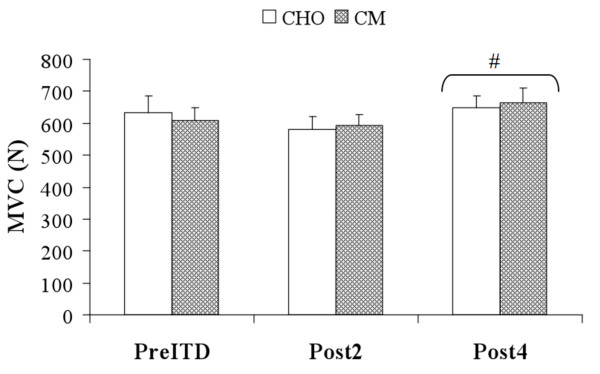
**MVC levels following Increased Training Duration**. Data reported are means/standard error. [# = significantly different (p < 0.05) than PreITD].

The effects of ITD and supplementation (CHO and CM) on recovery variables are included in Table 4 and Figures 1 &2. No significant treatment*time interactions were observed for any of the RM-ANOVA analyses (muscle soreness, MVC, MPSTEFS ratings). Significant (p < 0.05) main-effects for time were observed for muscle soreness and MVC. Serum CK levels rose significantly following PreITD, and CK was significantly different between treatments at the Post4 time-point (Figure 1). No significant between-treatment differences were observed for other recovery variables. Data from the soccer-specific performance tests are shown in Table 5. No significant differences were observed between treatment periods.

**Table 4:** Subjective Ratings of Muscle Soreness and Energy/Fatigue following Increased Training Duration

		Timepoint		
Recovery Variable	Treatment	Pre-ITD	Post2	Post4
Muscle Soreness*^#^ (mm)	CHO	43.2 ± 6.7	41.3 ± 6.3	48.8 ± 8.0
	CM	34.9 ± 6.4	37.3 ± 5.7	45.3 ± 7.5

Physical Energy (mm)	CHO	171.4 ± 14.8	178.6 ± 16.0	158.3 ± 19.1
	CM	162.6 ± 15.6	170.3 ± 19.0	166.7 ± 18.5

Physical Fatigue (mm)	CHO	133.3 ± 12.5	124.8 ± 13.9	115.8 ± 17.6
	CM	114.2 ± 13.5	126.4 ± 18.1	132.8 ± 19.5

Mental Energy (mm)	CHO	177.9 ± 12.9	166.8 ± 13.4	166.4 ± 19.4
	CM	172.4 ± 17	172.6 ± 18.1	164.3 ± 20.0

Mental Fatigue (mm)	CHO	135.8 ± 15.6	124.3 ± 12.5	125.8 ± 18.4
	CM	119.6 ± 16.4	121.2 ± 18.1	138.6 ± 19.8

Data reported are Mean ± SEM

* = significant main-effect for time (p < 0.05)

# = significant time-effect between Pre-ITD and Post4

**Table 5:** Performance Tests

	Treatment Period	
Performance Test	CHO	CM
T-Drill (s)	9.09 ± 0.13	9.06 ± 0.16
Vertical Jump (inches)	26.7 ± 1.0	26.7 ± 1.0

Data reported are Mean ± SEM

## Discussion

Training programs for competitive soccer players include activities of varying intensities, which have been shown to deplete muscle glycogen stores [25,26]. In addition, plyometric exercises such as vertical jumping, which are a common component of soccer training, have been associated with increased muscle soreness, elevated blood CK levels and impaired performance in subsequent exercise [27]. Thus, the utilization of post-exercise nutrition interventions that influence these variables could potentially affect recovery in soccer players. The purpose of this investigation was to assess the efficacy of CM as a post-exercise recovery beverage in soccer players, compared to a carbohydrate-only beverage. The recovery drinks were matched in total caloric content (504 kcal/serving), and both beverages contained carbohydrate in amounts that approached (CM: 1.1 g/kg) or exceeded (~CHO: 1.5 g/kg) levels associated with optimal post-exercise glycogen repletion [34,35]. Although few studies have investigated the specific effects of CM on post-exercise recovery, our findings can also be compared with studies investigating CHO+Pro recovery beverages, which contain carbohydrate and protein in similar proportions to CM.

Overall, the isocaloric CM and CHO supplements provided similar effects on markers of post-exercise recovery over the four-day period of ITD. No significant treatment*time interactions were observed for muscle soreness, ratings of energy/fatigue and muscle function (MVC). Similarly, there were no treatment effects on serum Mb. However, serum CK levels were significantly lower following four days of ITD with CM supplementation versus CHO supplementation. Numerous studies of CHO+Pro beverages have reported attenuated post-exercise plasma/serum CK levels after heavy endurance or resistance exercise [4,5,7-10], though this finding has not be observed in all studies [11,12]. The reduced CK levels observed in this investigation is also consistent with Cade et al. [24] and Luden et al. [6], who reported lower plasma CK levels with CHO+Pro ingestion over the course of multiple days of training in free-living swimmers and runners, respectively. Our findings similarly suggest that CM may attenuate blood CK levels in athletes performing heavy soccer training.

Plasma/serum CK is often used as a broad indicator of muscle damage. However, CK levels can be poorly correlated with direct measures of muscle damage or muscle function [36,37]. Thus, the practical significance of modestly lower serum CK levels (~115 U/L) with CM is not clear. This is particularly notable here because other measures of recovery, such as muscle soreness and Mb were not significantly different between treatments. Findings from other studies have reported mixed findings with respect to the influence of CHO+Pro on these variables. Some have reported attenuated muscle soreness ratings or Mb levels following heavy endurance [6-8,10,11] or resistance exercise [4,38], while others have reported no differences between treatments [12].

Though it cannot be concluded that recovery was different between treatments based on the CK data alone, other information from this study could suggest a potential tendency towards augmented recovery with CM. For example, increases in MVC over the four days of ITD were slightly greater with CM ingestion (53 ± 75 N) than with CHO (16 ± 93 N). This observation is consistent with findings from Valentine et al. [10], who reported that CHO+Pro ingestion improved muscle function versus CHO and placebo beverages following heavy endurance exercise. The difference in MVC levels between treatments in the present study was not statistically significant (p = 0.295), but may warrant investigation in future studies in light of the relatively small effect of our ITD protocol on symptoms of overreaching, as discussed below.

From a functional perspective, the most important measure of 'recovery' for athletes is performance in subsequent exercise. Some recent investigations have reported that CHO+Pro co-ingestion during/following heavy endurance exercise may improve subsequent exercise performance versus CHO [9,14-18]. However, a similar number of studies have reported no differences in subsequent performance between CHO and CHO+Pro recovery beverages [6-8,11,19-21]. Subsequent exercise performance was not assessed in the present study, as it was not possible to perform repeated sport-specific exercise testing within each training period without interfering significantly with the prescribed training programs from the coaching staff. However, sport-specific exercise tests (T-drill, vertical jump) were conducted within the ITD periods, and compared between treatments. Performance test results were virtually identical between treatment periods, suggesting that post-exercise CM consumption did not have a preferential effect on short-duration, high-intensity whole-body exercise performance versus CHO. Our findings suggest that isocaloric CHO and CM beverages provide similar effects on whole body exercise recovery during short periods of heavy soccer training.

Few studies have examined the specific effects of CM on recovery from heavy endurance-based exercise. Karp et al. [22] compared three recovery beverages consumed following a glycogen-depleting session of cycling intervals. In a time-to-exhaustion test performed four hours later, cyclists rode significantly longer with CM compared to a commercial CHO+Pro beverage, but had similar performances as compared to a commercial CHO beverage. These findings are difficult to interpret, as CM contained similar carbohydrate and protein amounts than the CHO+Pro beverage, but the CHO beverage contained no protein and less than half the carbohydrate and caloric content than CM. More recently, Thomas et al. [23] conducted a similar study, comparing isocaloric CM and CHO+Pro beverages and a CHO beverage comparable to that used by Karp et al. [22]. Time to exhaustion in the subsequent exercise bout was significantly longer with CM than either comparison beverage. Although the potential mechanisms for these findings are not clear, these studies support the potential efficacy of CM as a post-exercise recovery beverage following heavy endurance exercise.

The present study was designed to compare recovery beverages in free-living athletes within a collegiate team setting. Although this maximizes the generalizability of our findings for athletes, there were some relevant limitations to this design. Firstly, the free-living environment may have increased measurement error over the course of the study. Great care was taken throughout the study to insure that training/nutritional conditions were virtually identical between the two treatment periods. However, it is possible that activities outside the experimental protocols may have influenced the outcomes of the study. For example, four of the seventeen participants who completed the study were removed from statistical analyses (as described in Methods) due to large variations in baseline measurements (i.e. prior to ITD and beverage treatments), possibly due to activities outside of the study parameters. Six subjects failed to return completed dietary recall questionnaires, and thus we cannot be certain that nutrient intake did not vary between treatment periods for the entire sample. In addition, subjects were instructed to replicate the same dietary habits between treatment periods, but were not required to arrive at the laboratory in a fasted state. Thus, differences in nutrient timing between treatment periods could also have influenced some of the study outcomes.

Another limitation was the NCAA regulation limiting out-of-season practice time to a maximum of 8 hrs per week of 'athletically related activities' (NCAA Playing and Practice Limitations, Bylaw 17.1.5.2). As a result, it was not possible to implement an ITD period greater than 4 days in the present study. The prescribed training program was designed to increase daily training time by >25% per day between baseline and ITD periods (Table 1). However, due to adjustments in training plans to accommodate for inclement weather on two days (and maintain consistency between treatment periods), the ITD period increased daily training times by only 12% (Table 3). This training stimulus produced significant increases in muscle soreness ratings, and serum CK levels over the four-day period. However, MPSTEFS ratings and serum Mb were not significantly altered over time, and MVC actually improved over the four days of ITD. These findings suggest that the relatively modest increase in training volumes (combined with the daily consumption of post-exercise recovery beverages) may have been an inadequate stimulus to substantially impair muscle recovery. Without a relatively robust effect on these markers following exercise, it may be difficult to assess differences in recovery between treatments, especially with a relatively small sample of subjects, as described by Luden et al. [6]. This issue is particularly relevant with regards to our measurements of vertical jump performance. Byrne and Eston [33] reported that vertical jump performance declined to 90% of initial levels one day following muscle damaging exercise. However, their exercise protocol produced elevations in CK that were approximately 3-4 times greater than the present study. Because our vertical jump device assessed only 0.5 inch increments, our instrument potentially lacked the sensitivity to detect realistic changes in vertical jump height. Other investigators have reported significant decrements in physical performance, fatigue and/or muscle soreness following periods of ITD [3,39]. However, these studies provided 8-11 days of ITD (and relatively low post-exercise carbohydrate intake), which represented a much greater alteration in training stimulus than the present study. Thus, it may be worthwhile for future researchers to investigate the efficacy of CM during longer, more demanding periods of ITD.

Due to the practical restrictions of studying collegiate athletes, it was also not possible to add a placebo trial to the present study design. This prevented us from establishing the direct effects of the ITD period, independent of supplementation. Recovery beverages were provided immediately post-exercise, and both contained high doses of carbohydrate (>1.1 g/kg). As a result, both beverages probably produced high rates of post-exercise glycogen resynthesis [40], and potentially sustained muscle recovery and performance levels to a greater degree than if inadequate carbohydrate were provided [3,39]. However, the relative efficacy of the 'control' beverage in this study (CHO) cannot be quantified without a placebo trial for comparison.

## Conclusions

In summary, post-exercise CM supplementation resulted in significantly lower serum CK levels following four days of heavy soccer training. However, other measurements of muscle recovery were generally similar between treatment beverages, and there were no differences in whole-body exercise performance between treatments. Thus, exercise recovery during short-term periods of heavy soccer training appears to be similar when isocaloric CM and CHO beverages are consumed post-exercise. It is possible that potential differences between treatments could be magnified by a greater training stimulus. Thus, it is recommended that future studies perform similar comparisons during training periods that involve greater increases in training volumes over longer periods of time.

## Competing interests

MJS has served as a member of an advisory committee for the National Dairy Council, and has received fees and travel reimbursement for work related to this role.

## Authors' contributions

SFG participated as the lead author and participated in study design, screening and recruitment, data collection, analysis and interpretation, and final draft of the manuscript. MJS, acting as senior thesis advisor, participated in study design, screening and recruitment, data collection, analysis and interpretation, and final draft of the manuscript. CWM participated in data collection, analysis and interpretation, and editing of the manuscript. RWM participated in data collection and interpretation, and editing of the manuscript. CJW, acting as a thesis advisor, assisted with study design, data analysis and interpretation, and editing of the manuscript. MKT, acting as a thesis advisor, assisted with study design, data analysis and interpretation, and editing of the manuscript. All authors have read and approved the final draft of this manuscript.
